# Ageing is associated with molecular signatures of inflammation and type 2 diabetes in rat pancreatic islets

**DOI:** 10.1007/s00125-015-3837-8

**Published:** 2015-12-23

**Authors:** Ionel Sandovici, Constanze M. Hammerle, Wendy N. Cooper, Noel H. Smith, Jane L. Tarry-Adkins, Benjamin J. Dunmore, Julien Bauer, Simon R. Andrews, Giles S. H. Yeo, Susan E. Ozanne, Miguel Constância

**Affiliations:** Metabolic Research Laboratories and MRC Metabolic Diseases Unit, Wellcome Trust-Medical Research Council Institute of Metabolic Science, University of Cambridge, Addenbrooke’s Hospital, Cambridge, CB2 OQQ UK; Department of Obstetrics and Gynaecology, University of Cambridge, The Rosie Hospital, Robinson Way, Cambridge, CB2 0SW UK; Centre for Trophoblast Research, University of Cambridge, Cambridge, UK; Cambridge Genomic Services, Department of Pathology, University of Cambridge, Cambridge, UK; Bioinformatics Group, The Babraham Institute, Cambridge, UK; National Institute of Health Research, Cambridge Biomedical Research Centre, Cambridge, UK

**Keywords:** Ageing, DNA methylation, Epigenetics, Inflammageing, Pancreatic islets, Type 2 diabetes

## Abstract

**Aims/hypothesis:**

Ageing is a major risk factor for development of metabolic diseases such as type 2 diabetes. Identification of the mechanisms underlying this association could help to elucidate the relationship between age-associated progressive loss of metabolic health and development of type 2 diabetes. We aimed to determine molecular signatures during ageing in the endocrine pancreas.

**Methods:**

Global gene transcription was measured in pancreatic islets isolated from young and old rats by Ilumina BeadChip arrays. Promoter DNA methylation was measured by Sequenom MassArray in 46 genes that showed differential expression with age, and correlations with expression were established. Alterations in morphological and cellular processes with age were determined by immunohistochemical methods.

**Results:**

Age-related changes in gene expression were found at 623 loci (>1.5-fold, false discovery rate [FDR] <5%), with a significant (FDR < 0.05) enrichment in genes previously implicated in islet-cell function (*Enpp1*, *Abcc8*), type 2 diabetes (*Tspan8*, *Kcnq1*), inflammatory processes (*Cxcl9*, *Il33*) and extracellular matrix organisation (*Col3a1*, *Dpt*). Age-associated transcriptional differences negatively correlated with promoter DNA methylation at several loci related to inflammation, glucose homeostasis, cell proliferation and cell–matrix interactions (*Il33*, *Cxcl9*, *Gpr119*, *Fbp2*, *Col3a1*, *Dpt*, *Spp1*).

**Conclusions/interpretation:**

Our findings suggest that a significant proportion of pancreatic islets develop a low-grade ‘chronic’ inflammatory status with ageing and this may trigger altered functional plasticity. Furthermore, we identified changes in expression of genes previously linked to type 2 diabetes and associated changes in DNA methylation that could explain their age-associated dysregulation. These findings provide new insights into key (epi)genetic signatures of the ageing process in islets.

**Electronic supplementary material:**

The online version of this article (doi:10.1007/s00125-015-3837-8) contains peer-reviewed but unedited supplementary material, which is available to authorised users.

## Introduction

The normal process of ageing is a complex phenomenon associated with a variety of physiological alterations in the function of cells and organs. These alterations are thought to be major determining risk factors for many traditionally adult-onset diseases, including type 2 diabetes [1]. The progressive decline with age of pancreatic islet function in humans, in the presence of increasing insulin resistance, is thought to be a key factor contributing to the pathophysiology of type 2 diabetes [2]. For example, age-associated impairment in insulin secretion has been demonstrated in rodents as well as in humans [3]. When such insulin secretory defects are superimposed over an increased need for insulin as observed in old age, impaired glucose tolerance and type 2 diabetes may ensue. However, the causes of age-related degenerative changes in pancreatic islets remain poorly defined. Studies of cellular and metabolic processes have led to theories that implicate increased oxidative stress, impaired cell proliferation and apoptosis as causative factors [4].

More recently, epigenetic processes have been suggested to underpin the loss of plasticity of cells that occurs during ageing [5]. DNA methylation is a major epigenetic modification, with essential roles in gene regulation and chromatin organisation. Ageing has been correlated with both DNA methylation gain [6] and loss [7], in a locus-dependent manner. Moreover, there is a strong tissue specificity of age-associated alterations in DNA methylation [8]. Altered DNA methylation has been reported in pancreatic islets of type 2 diabetic patients compared with non-diabetic individuals [9, 10]. Cell-type composition and function of pancreatic islets change in response to physiological alterations with age [11–15]. In the current study, we used isolated rat pancreatic islets to study ageing in the context of the complex cell-type interactions that characterise the endocrine pancreas. We profiled genome-wide gene expression in pancreatic islets at two time points—young (3 months of age) vs old (15 months of age). We then established the extent to which differential expression with age could be explained by altered DNA methylation by screening promoter methylation at a number of selected genes. Our findings revealed age-associated gene signatures of ‘inflammageing’ and type 2 diabetes. We suggest that the detrimental events that occur during ageing and increase the risk of impaired glucose homeostasis might be triggered by a low-grade ‘chronic’ inflammatory status of the endocrine pancreas.

## Methods

### Animals

All procedures were conducted in accordance with the 1986 British Home Office Animals Act. Each sample was composed of islets isolated from two to four male Wistar rats (Charles River, Margate, Kent, UK), which were killed at 3 months or 15 months (±3 days) of age after overnight fasting (*n* = 4–10 samples per measurement).

### Islet isolation, RNA extraction and purity assessment

**Fig. 1:**
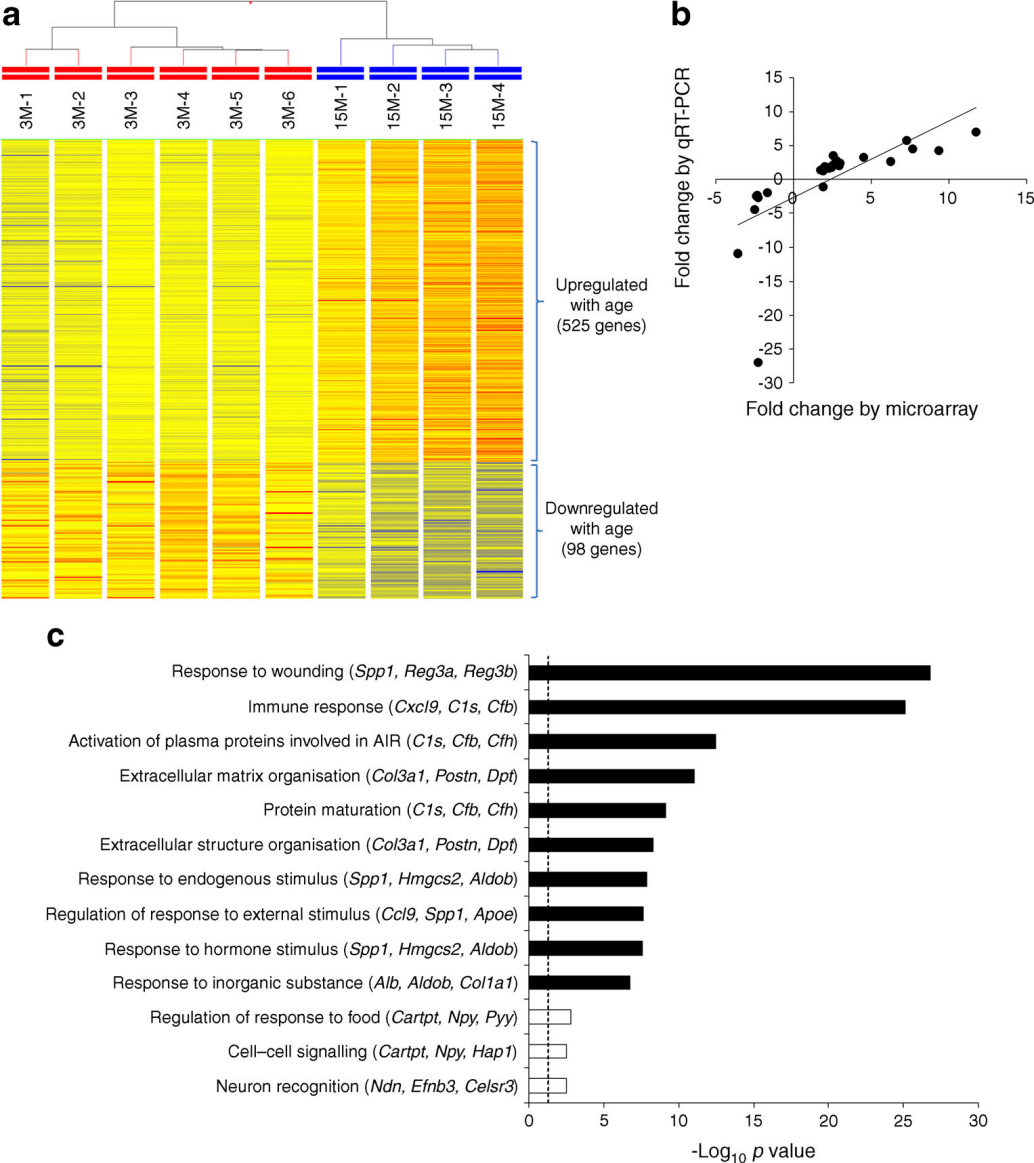
Genes with age-related changes in mRNA expression in rat islets. (**a**) Heat map depicting genes differentially expressed between young and old islets. (**b**) Biological validation by qRT-PCR for 26 genes (*R* = 0.6655, *p* = 4.14 × 10^−4^). (**c**) Top-scoring biological processes enriched in genes upregulated with age (black bars) or downregulated with age (white bars) (see also ESM Table 5). Between parentheses are indicated three genes with the highest age-related fold expression changes. The dotted line corresponds to *p* = 0.05. AIR, acute inflammatory response

Isolation of rat islets and RNA extraction was performed as described [16]. Purity of rat islets samples was assessed by quantitative (q)RT-PCR quantification of the acinar-specific genes *Amy2* and *Prss1* (see electronic supplementary material [ESM] Fig. 1).

### Expression microarray analysis

Array profiling was performed using the RatRef-12 Expression BeadChips (Illumina, Little Chesterford, UK). Analysis of microarray data was performed using GeneSpring GX 12.1 (Agilent, Santa Clara, CA, USA). Signal intensity levels across arrays were normalised to the 75th percentile, with baseline transformation to the median intensity of all samples. Genes with signal intensities <50 after normalisation were classified as non-expressed. Hierarchical clustering was performed using a Euclidean distance metric and a centroid distance rule. Criteria used for filtering ‘ageing genes’ were: false discovery rate (FDR) <0.05 from an unpaired *t* test following Benjamini–Hochberg correction for multiple testing and 1.5-fold age-related change in expression. Genes with age-related expression changes in rat islets that had previously been associated with type 2 diabetes or islet function (ESM Table 1) were identified by a keyword-based search in OMIM (Online Mendelian Inheritance in Man), genome-wide association study (GWAS) catalogue and PubMed.

### qRT-PCR validation of expression microarrays

RNA (1 μg, *n* = 10 samples per group) was used to synthesise cDNA with the RevertAid H Minus Reverse Transcriptase kit (Fermentas, Loughborough, UK). Quantification of gene expression was performed using the ABI Prism 7900 system (Applied Biosystems, Foster City, CA, USA) and primers for SYBR Green or TaqMan probes (Applied Biosystems) (ESM Table 2). Fold changes in gene expression were calculated using the REST 2009 programme (Qiagen, Manchester, UK) and normalised against *Ppia*, *Taf2* and *Hmbs*, identified as good internal controls from the microarray data.

### Functional annotation and enrichment analysis

This was performed using DAVID v6.7 (Database for Annotation, Visualization and Integrated Discovery; http://david.abcc.ncifcrf.gov/, accessed 16 July 2013) to assess whether there was enrichment for genes implicated in particular biological processes (background used: RatRef-12_V1_0_R4_11222119_A). Enriched gene ontology (GO) terms with an FDR < 0.05 were considered significant. These terms were then clustered semantically using the REViGO (Reduce and Visualize GO) server [17], which removes redundancy. The results obtained by REViGO were ordered according to log_10_*p* values.

### DNA methylation analysis using bisulfite MassArray (Sequenom)

Genomic DNA (1 μg) was extracted from islets or BRIN-BD11 cells and was treated with sodium bisulfite (EZ DNA methylation kit; Zymo, Irvine, CA, USA). Primer pairs were designed using EpiDesigner (Sequenom, Hamburg, Germany) (ESM Table 2). MassArray analysis was performed in triplicate according to manufacturer’s instructions (Sequenom).

We measured DNA methylation levels at the promoter regions of 46 genes that displayed age-related mRNA expression changes. We included genes identified as hits for type 2 diabetes by GWAS (*Itgb6*, *Kcnq1*, *Tspan8*), genes differentially methylated between diabetic and control islets in humans (*Pyy*, *Tspan4*, *Lgals2*, *Spp1*), genes implicated in monogenic forms of diabetes (*Abcc8*) and genes that belong to the enriched pathways described in Fig. 1c (for complete list see ESM Table 3).

### Pharmacological treatment

BRIN-BD11 cells (GlaxoSmithKline, Stevenage, UK) were cultured as described [16]. Cells (10^5^/cm^2^) were seeded in T-75 flasks 24 h before the start of the treatment, which consisted of six daily administrations of fresh media containing 100 μmol/l zebularine (Sigma-Aldrich, St Louis, MO, USA) dissolved in DMSO, or DMSO only as vehicle control. The analysis was performed in nine of the 14 genes with inverse correlation between DNA methylation and gene expression. The remaining five genes could not be analysed, as their methylation levels were either very low or the genes were not expressed in BRIN-BD11 cells in the presence or absence of zebularine.

### Histological and immunohistochemical staining

Staining for β-galactosidase was performed on frozen sections using the Senescence Cells Histochemical Staining kit (Sigma-Aldrich). Sirius Red staining was performed on paraffin sections as previously described [18].

All immunohistochemistry and immunofluorescence staining was performed on paraffin sections (details are provided in ESM Table 2). Slides without primary antibodies were used to assess specificity of each staining (data not shown).

For histochemistry and immunohistochemistry staining slides were scanned using a Hamamatsu NanoZoomer scanner then examined using the NDP.view software (Hamamatsu, Japan). All islets from at least one section (*n* = 4 biological replicates per group) were inspected and scored by a researcher blinded to the sample’s identity.

For immunofluorescent stainings image acquisition was performed using a LSM510 Meta confocal laser scanning microscope (Carl Zeiss, Jena, Germany) and the ZEN 2009 software (http://www.zeiss.com/microscopy/en_de/products/microscope-software.html). At least ten randomly selected islets were imaged for each section (*n* = 4 biological replicates per group). For glucagon/insulin double staining total glucagon and insulin surface areas were measured using the Visiopharm software (Visiopharm, Hoersholm, Denmark). For regenerating islet-derived 3α (REG3A) and chemokine (C-X-C motif) ligand 9 (CXCL9) stains the intensity of staining was visually scored by three viewers blinded to the sample’s identity and averages were used for statistical analysis.

### Statistical analyses

For qRT-PCR data statistical analysis was performed using the REST 2009 programme (Qiagen, Manchester, UK). All other statistical tests were performed using the GraphPad 5.04 software (http://www.graphpad.com). A *p* value of <0.05 was considered statistically significant.

## Results

### Age-related gene expression changes in rat islets

We generated microarray datasets of islets isolated from 3-month-old (3M) and 15-month-old (15M) male rats, using Illumina’s Expression BeadChips, which interrogate ~21,900 genes. Information on the preparation and purity of the rat islet samples is summarised in the Methods and ESM Fig. 1. We identified 623 genes out of the 16,885 genes expressed in islets, which had significant age-related transcriptional changes (>1.5-fold, FDR < 0.05). There was a strong bias for age-associated upregulation (525 genes vs 98 downregulated genes; sign test *p* = 2.76 × 10^−65^). The complete list of genes with significant age-related changes in expression is depicted as a heat map in Fig. 1a and is provided in full in ESM Table 4. These 623 genes include 44 that have been previously implicated in beta cell function and/or linked to type 2 diabetes through GWAS (ESM Table 1). For microarray validation, expression of 26 genes (shown in red in ESM Table 4, and including six of those listed in ESM Table 1 [*Cartpt*, *S100a6*, *Ptger3*, *Reg3a*, *Pyy* and *Npy*]) was measured by qRT-PCR. We found a strong positive correlation between microarray and qRT-PCR measurements (*R* = 0.6655, *p* = 4.14 × 10^−4^ Fig. 1b).

Using DAVID functional annotation, followed by REViGO filtering (see Methods), we found that genes upregulated with age were significantly enriched for cellular processes related to immune and inflammatory responses and extracellular matrix organisation. According to the criteria used there were only three biological processes significantly enriched in genes downregulated with age: regulation of response to food, cell–cell signalling and neuron recognition (see Fig. 1c for the top biological processes influenced by age and ESM Table 5 for the comprehensive list).

### Age-related changes in DNA methylation of differentially expressed loci

To test the hypothesis that the mRNA expression changes with age were associated with alterations in DNA methylation levels at promoter regions we used bisulfite MassArray assays [19] to measure DNA methylation levels at 46 loci (36 showed upregulation with age and ten displayed downregulation with age; see Methods for selection criteria and ESM Table 3 for complete list).

**Fig. 2:**
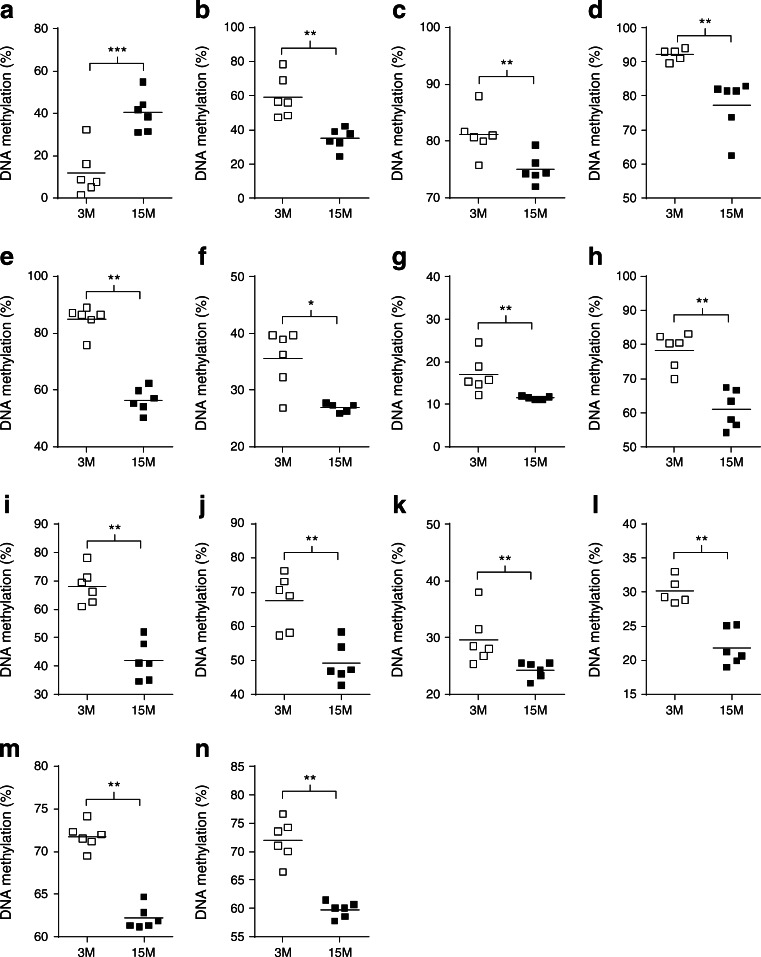
Genes with significant age-related differences in DNA methylation (>5%) at promoter regions that demonstrated an inverse relationship with mRNA expression levels: *Gpr119* (**a**), *Fbp2* (**b**), *S100a4* (**c**), *Il33* (**d**), *Pah* (**e**), *Lgals1* (**f**), *Mgst1* (**g**), *Dpt* (**h**), *C4b* (**i**), *Col3a1* (**j**), *Prss35* (**k**), *Tf* (**l**), *Spp1* (**m**) and *Cxcl9* (**n**). White squares, 3M samples; black squares, 15M samples. Horizontal bars indicate mean values. *p < 0.05, **p < 0.01 and ***p < 0.001 for 15M vs 3M, by Mann–Whitney tests

The methylation status of 26 of the loci studied (56.5%) changed with age (ESM Table 3). However, only 19 showed an absolute difference in methylation above 5% (averaged across all CpG sites), with a maximum DNA methylation change of 28.4% (ESM Table 3). Of these 19 genes, 14 demonstrated a direction of change consistent with the age-associated effects on transcription (i.e. loss or gain of methylation correlating with increased or decreased gene expression, respectively) (Fig. 2, ESM Fig. 2 and ESM Table 3). Notably, several genes with altered DNA methylation belong to enriched pathways shown in Fig. 1c (i.e. inflammation [*Il33*, *Cxcl9*], glucose homeostasis [*Gpr119*, *Fbp2*] and extracellular matrix organisation [*Col3a1*, *Dpt*, *Spp1*]) (ESM Table 5).

To address the relationship between methylation and transcription we used the DNA methylation inhibitor zebularine [20] in BRIN-BD11 insulin-secreting cells. After 6 days of treatment with 100 μmol/l zebularine, we found significant reductions in DNA methylation at the promoter regions of all of the nine loci tested (ESM Fig. 3a; see Methods for exclusion criteria of five loci out of the 14). We then measured by qRT-PCR the corresponding mRNA levels and found that zebularine treatment led to a substantial increase in expression of five genes (*Fbp2*, *S100a4*, *Lgals1*, *Tf* and *Cxcl9*), consistent with demethylation leading to increased transcription. In contrast, zebularine treatment had no effect on the expression of the remaining four genes (*Il33*, *Pah*, *C4b* and *Col3a1*) (ESM Fig. 3b).

We also measured DNA methylation of LINE-1 transposable retroelements, which constitute almost 23% of the rat genome [21]. We found a significant 5.5% loss of DNA methylation with age (ESM Table 3).

### Tissue and cellular changes associated with ageing

As shown previously, DAVID analysis revealed that a number of cellular processes related to wound healing, immune responses and extracellular matrix organisation (Fig. 1c) are altered between young and aged islets. These findings prompted us to investigate signs of inflammation and fibrosis within the islets of aged rats.

**Fig. 3:**
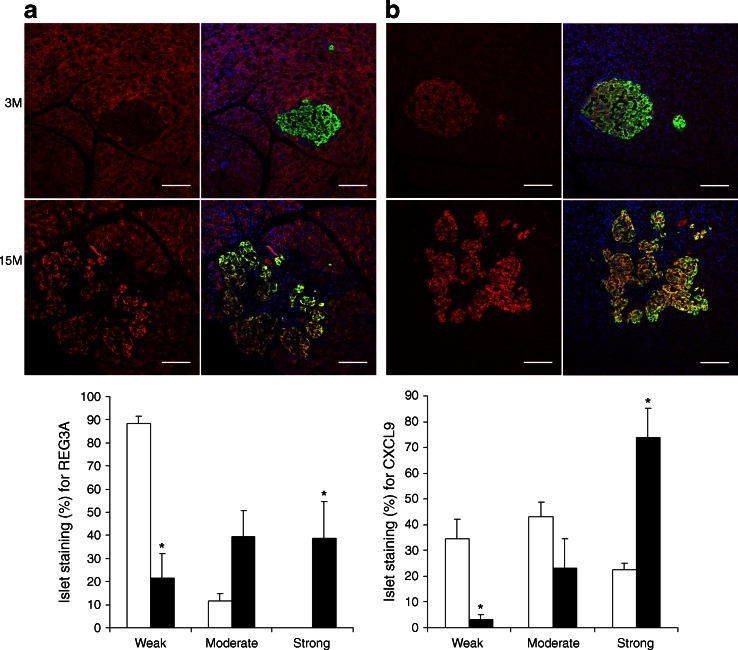
Immunofluorescence analysis of candidate proteins that exhibit age-related changes in mRNA expression in rat islets (red channel): REG3A (**a**) and CXCL9 (**b**). For both figure parts representative islets are depicted, scored as weakly stained (3M) and strongly stained (15M). The composite images visualise pancreatic beta cells stained with insulin (green) and nuclei stained with DAPI (blue). Scale bars, 100 μm. The histograms show the quantification for each stain (white bars, 3M; black bars, 15M). Data represent the mean of *n* = 4 samples per group; error bars represent SEM; *p < 0.05 for 15M vs 3M, by Mann–Whitney tests

First, we confirmed by qRT-PCR that a number of genes corresponding to the enriched biological processes were differentially expressed in an extended cohort of samples (13 genes highlighted in yellow in ESM Table 4). At the protein level we showed increased expression of two of the top five genes upregulated with age at the mRNA level, *Reg3a* and *Cxcl9* (Fig. 3), related to wound response and immune response, respectively.

**Fig. 4:**
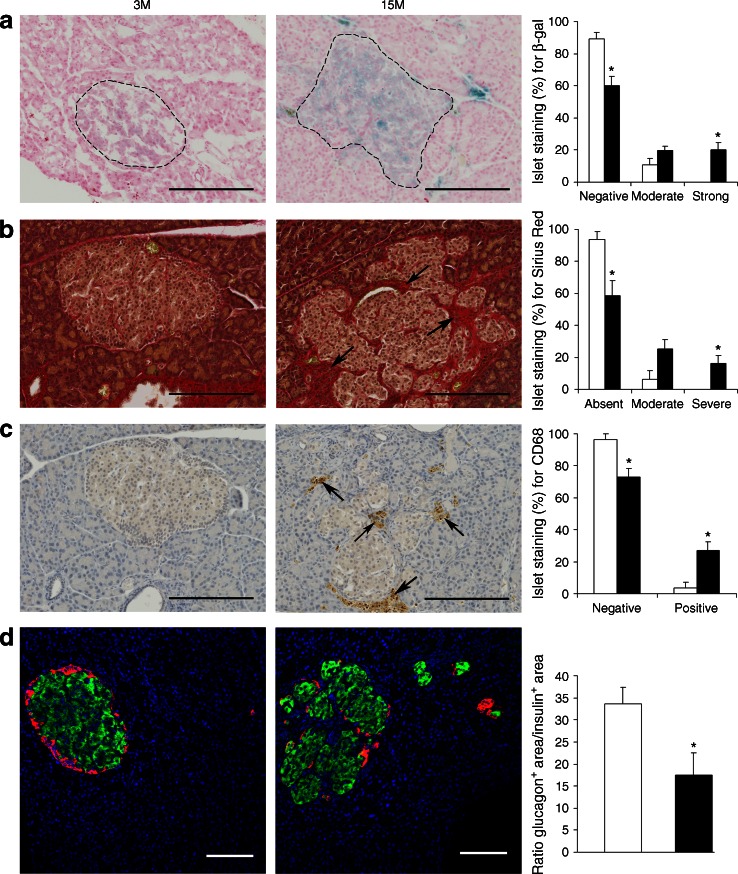
Histochemical and immunohistochemical analysis of 3M and 15M rat pancreases. (**a**) β-Galactosidase (β-gal) staining for cell senescence. The dotted lines outline pancreatic islets. (**b**) Sirius Red staining for collagen. Arrows indicate deposition of collagen. (**c**) Immunohistochemical staining for the macrophage marker CD68. Arrows indicate accumulation of macrophages. (**d**) Double immunostaining for glucagon (red) and insulin (green). Scale bars, 200 μm (**a**–**c**) and 100 μm (**d**). The histograms show the quantification for each stain (white bars, 3M; black bars, 15M). Data represent the mean of *n* = 4 samples per group; error bars represent SEM; *p < 0.05 for 15M vs 3M, by Mann–Whitney tests

Second, to investigate further the extent to which islet morphology and cellular function is changed with age, we evaluated markers of cellular senescence, macrophage infiltration, fibrosis and beta-to-alpha cell ratios, by (immuno)/histochemical staining in sections of young and old islets and performed a semiquantitative analysis (Fig. 4). Strong staining for β-galactosidase, a known marker of cellular senescence [22], was seen in ~20% of old islets, but was undetectable in any of the islets from young rats (Fig. 4a). Deposition of collagen, measured by Sirius Red staining, was observed in young islets at a low frequency (~7% of islets with moderate degree of collagen staining) and increased with age (~42% of islets with moderate to severe staining) (Fig. 4b). Macrophage infiltration, assessed by CD68 immunostaining, a pan-marker of all macrophage lineages [23], was almost exclusively found in old islets (at a frequency of ~27%) (Fig. 4c). To further phenotype the infiltrating macrophages we stained for nitric oxide synthase 2 (NOS2, also denoted as iNOS), a marker of the ‘classically activated’, proinflammatory M1 macrophages [24] (ESM Fig. 4a), and for arginase 1 (ARG1), a marker for the ‘alternatively-activated’ anti-inflammatory M2 macrophages [24] (ESM Fig. 4b). Both types of macrophages were present in ~24% of the islets from old rats. Glucagon and insulin staining was performed to assess whether aged pancreatic islets maintain the proportion of alpha and beta cells seen in young islets (Fig. 4d). Our results suggest that the known age-related increase in beta cell mass [25] was not accompanied by a concomitant increase in alpha cell mass, leading to a difference in cell composition between islets from young rats and those from old rats (Fig. 4d).

## Discussion

It is well established that ageing is associated with the functional decline of endocrine organs and therefore a risk factor for metabolic conditions, such as type 2 diabetes, in later life. Little work has been done to define the effects of ageing on the transcriptome of the endocrine pancreas and how epigenetic mechanisms could contribute to these effects. In this study we analysed the dynamics of gene transcription during ageing of the endocrine pancreas and measured promoter DNA methylation at a selected number of loci with age-associated transcriptional changes. We found age-related molecular signatures of ‘inflammageing’ and ‘type 2 diabetes’, with parallel changes in DNA methylation of genes contributing to this effect. We propose that these (epi)genetically regulated ‘signature’ genes represent novel mediators of the age-associated decline in islet-cell function.

We identified 3.7% genes within the islet transcriptome that were differentially expressed between young and old islets, by 1.5-fold or more, with the majority (84.3%) showing increases in expression with age. Pathway analysis revealed an enrichment of upregulated genes implicated in inflammation and immune responses (e.g. genes encoding homeostatic and inflammatory chemokines, interferon and interleukin, complement genes and genes encoding MHC molecules, which are involved in antigen presentation), suggestive of age-associated increases in endocrine pancreas inflammation (or inflammageing). Inflammageing, a term used to describe an imbalance between inflammatory and anti-inflammatory networks that causes lifelong antigenic burden and exposure to damaging agents, is thought to be a major driving force for frailty and common age-related pathologies [26]. Our findings lead us to propose that inflammageing may contribute to the known age-associated decline in beta cell function and consequential increase in type 2 diabetes risk. There is a substantial lack of functional studies that link those processes to endocrine function. Importantly, a transgenic mouse with increased expression of *Ccl2* (encoding chemokine [C-C motif] ligand 2) in their beta cells developed diabetes [27]; this is consistent with our observed age-related upregulation of *Ccl2* being detrimental for endocrine pancreatic function. Our findings are also consistent with studies showing that cells undergoing senescence start secreting a large number of pro-inflammatory cytokines and chemokines, as part of the so-called ‘senescence-associated secretory phenotype’ (SASP) [28]. We identified several genes that encode SASP proteins and that were upregulated with age in rat islets, including *Igfbp6*, *Mmp12*, *Mmp14*, *Timp2* and *Fn1*.

Many other genes that exhibited transcriptional dysregulation with age have been linked previously with type 2 diabetes (ESM Table 1). For example, microarray studies performed on human pancreatic islets isolated from patients with type 2 diabetes and glucose-tolerant controls identified, among other changes, overexpression of *ENPP1*, *TSPAN4*, *TSPAN8*, *CSRP1*, *REG3A* and *REG3G* [29, 30], genes that were also upregulated with age in our study. Some of the genes identified as being differentially expressed with age have been previously implicated in type 2 diabetes through GWAS [31]. These include *TSPAN8*, *KCNQ1* and *ITGB6* loci (upregulated with age in rat islets) and some of these polymorphisms have been predicted to affect beta cell function. Other genes expressed differentially with age have been previously implicated in endocrine pancreas function (ESM Table 1). These genes could therefore represent novel mediators of the age-associated decline in islet cell function.

As expected from the nature of this study, we found a number of gene expression ‘signatures’ of the ageing process. Fifteen of the genes upregulated with age (*C1qa*, *C1qb*, *Ctss*, *S100a6*, *Lgals3*, *Mgst1*, *S100a4*, *Fcgr2a*, *Gbp2*, *Il33*, *Litaf*, *Serping1*, *Spp1*, *Laptm5* and *Txnip*) had similar age-related gene expression changes to the ones reported in a recent meta-analysis of mouse, rat and human tissues (over ten tissues but not including pancreatic islets) [32, 33]. These genes therefore appear to be part of a common signature of ageing across tissues that include a total of 73 genes [32, 33].

Age-related changes in DNA methylation in the endocrine pancreas have not been reported prior to this study. We focused our analysis on a restricted number of loci (*n* = 46), based on the microarray differential expression analysis, with the aim of establishing correlations between DNA promoter methylation and transcriptional activity. Globally, 19 of the 46 loci analysed (41%) showed absolute differences in methylation of between 5% and 28%. Fourteen of the 19 differentially methylated genes (74%) demonstrated a direction of change consistent with differences in expression, suggesting that DNA methylation may contribute to the transcriptional changes. Notably, 13 of these 14 loci reflected hypomethylation with age and were associated with increased expression. These changes were observed at loci related to ‘inflammageing’ and endocrine function. Global analysis of DNA methylation, as measured by methylation levels of LINE-1 repeats, further suggested that hypomethylation might be an epigenetic hallmark of ageing in rat islets. Importantly, a major preponderance for loss of DNA methylation has been observed recently in pancreatic islets of type 2 diabetes patients [9, 10].

Our current study has one important limitation, which is the use of islet cells instead of purified cell types. A clear advantage of using islet cells is that this reflects the physiology of the endocrine pancreas and takes into account the milieu of insulin-secreting cells. However, the transcriptomic and epigenetic data analyses are made more complex given potential confounding effects brought about by morphology and cell-type composition changes with age. In this study, we found clear evidence for cellular senescence, collagen deposition and macrophage infiltration in a significant proportion of aged islets (affecting ~20%, ~ 42% and ~27% of all islets, respectively). Associated with these morphological changes, 11 collagen genes were upregulated in our dataset (ESM Table 4) as was the macrophage marker CD68 (Fig. 4c). Importantly, age-related morphological changes in the pancreatic islets, such as beta cell hyperplasia and intra-insular fibrosis with accumulation of macrophages, have been recognised to occur spontaneously in aged (predominantly male) rats fed ad libitum [14, 15]. Similar morphological changes have also been reported in several rat models of diabetes and in obese rat strains [34–36]. Pancreatic islet pathology is highly variable in patients with type 2 diabetes, with moderate changes in beta cells, islet fibrosis and amyloidosis being commonly reported [14]. These morphological changes are likely to have an impact on DNA methylation patterns. Whether the altered DNA methylation seen in this current study reflects cell-type composition differences or relates to transcriptional activity only, or a combination of both, is difficult to ascertain. Our studies using a demethylating agent in an insulin-secreting cell line suggest that loss of DNA methylation might be responsible, at least in part, for the observed transcriptional changes at five loci (*Fbp2*, *S100a4*, *Lgals1*, *Tf* and *Cxcl9*). *Cxcl9* is of particular interest as it is upregulated at both the mRNA (ranking as second highest gene upregulated with age in our dataset; ESM Table 4) and protein level (Fig. 3b) and is hypomethylated with age. CXCL9 is a small cytokine that has been shown to play an anti-fibrotic role in experimental chronic pancreatitis in rats and is a promising therapeutic target in pancreatic fibrosis [37]. *S100a4* (encoding S100 calcium binding protein a4) has been shown to increase with age in a number of tissues [32]. However, it has not previously been established whether this is associated with changes in DNA methylation. Whether or not the age-related changes in DNA methylation observed at these loci are directly linked to the increased risk for type 2 diabetes will need to be established in future studies.

In conclusion, our study demonstrates that ageing leads to a relatively small number of transcriptional alterations in the endocrine pancreas, associated with an ‘inflammageing’ phenotype and type 2 diabetes signatures. The only other microarray analysis investigating the impact of ageing on the pancreatic islet transcriptome (interrogating ~13,000 mouse genes) [38] did not reported the same findings (there are several important differences between the two studies, including species, age time points, transcriptomic coverage and islet cell purity) and did not link the transcriptome to changes in epigenetic alterations. A very recent study of beta cell ageing in the mouse identified ~6,000 differentially expressed genes and over 14,000 differentially methylated regions, the majority of which were located more than 1 kb distal to transcriptional start sites. This study was conducted in sorted beta cells, therefore the impact of cellular processes described in our study (e.g. macrophage infiltration, collagen deposition) during ageing were missed [39]. We suggest that age-associated low-grade inflammation is linked with cellular (e.g. fibrosis) and metabolic dysfunction (e.g. de-regulation of genes involved in type 2 diabetes pathogenesis). These findings indicate that the local pancreatic environment (e.g. inflammation and fibrosis), in addition to systemic effects that occur with age, may be a major cause for the age-dependent decline of endocrine function. Furthermore, we propose that alterations in promoter DNA methylation can modulate these processes, although causal relationships need to be established.

Future studies aimed at assessing the biological roles of the genes highlighted in this study, using in vitro assays and/or in vivo animal models, have the potential to expand our knowledge on type 2 diabetes pathogenesis. It will be important to investigate whether the same mechanisms reported in this study are also observed in aged human islets.

## Electronic supplementary material

ESM Table 1 (PDF 22 kb)

ESM Table 2 (XLS 52 kb)

ESM Table 3 (PDF 10 kb)

ESM Table 4 (XLS 146 kb)

ESM Table 5 (XLS 305 kb)

ESM Fig. 1 (PDF 100 kb)

ESM Fig. 2 (PDF 1212 kb)

ESM Fig. 3 (PDF 280 kb)

ESM Fig. 4 (PDF 631 kb)

## Abbreviations

3M: 3-Month-old
15M: 15-Month-old
CXCL9: Chemokine (C-X-C motif) ligand 9
DAVID: Database for Annotation Visualization and Integrated Discovery
FDR: False discovery rate
GO: Gene ontology
GWAS: Genome-wide association study
OMIM: Online Mendelian Inheritance in Man
qRT-PCR: Quantitative reverse transcription PCR
REG3A: Regenerating islet-derived 3α
REViGO: Reduce and visualize GO
SASP: Senescence-associated secretory phenotype
