# Supplementary material for: Ageing is associated with molecular signatures of inflammation and type 2 diabetes in rat pancreatic islets
Source: Diabetologia. 2015 Dec 23;59:502–11. doi: 10.1007/s00125-015-3837-8 (PMC4742511; doi:10.1007/s00125-015-3837-8)
Supplement: Supplementary file 1 — (PDF 22 kb) [file 125_2015_3837_MOESM1_ESM.pdf]

ESM Table 1 Genes with age-related expression changes in rat islets and previously associated with T2D traits or islet function

**Symbol**	**Gene name**	**Fold change**	**Significance**	**Reference (PMID)**
Reg3a	Regenerating islet-derived 3 alpha	+7.6	Increased expression in human pancreatic beta cells of individuals with T2D	20644627
Reg3g	Regenerating islet-derived 3 gamma	+5.4	Increased expression in human pancreatic beta cells of individuals with T2D	20644627
Onecut1	One cut homeobox 1	+4.2	Required for islet beta cell development; postnatal expression alters beta cell function	17130469
Ptf1a	Pancreas specific transcription factor, 1a	+3.5	Role in early pancreas development; mutations in *PTF1A* cause pancreatic agenesis associated with permanent neonatal diabetes mellitus	15543146<br>18519637
Kcnq1	Potassium voltage-gated channel, KQT-like subfamily, member 1	+2.9	GWAS association with T2D	18711367
Hmox1	Heme oxygenase (decycling) 1	+2.9	Longer (GT)n repeats in the gene promoter are associated with increased risk for T2D	20682519
Cyp2e1	Cytochrome P450, family 2, subfamily e, polypeptide 1	+2.7	Human pancreatic beta cells expressing *CYP2E1* are more susceptible to DNA damage and cytotoxicity	15242818
Hsd11b1	Hydroxysteroid 11-beta dehydrogenase 1	+2.6	Expressed by the pancreatic alpha cells; regulates insulin and glucagon secretion	18779947
Tspan8	Tetraspanin 8	+2.6	GWAS association with T2D; increased expression in human pancreatic beta cells of individuals with T2D	18372903<br>20644627
Tmed6	Transmembrane emp24 protein transport domain containing 6	+2.6	Expression restricted to pancreatic islets, with higher levels in alpha cells; expression reduced in diabetic Goto-Kakizaki rats	22129529
Ptger3	Prostaglandin E receptor 3 (subtype EP3)	+2.5	Increased expression in islets of BTBR diabetic mice; negatively regulates glucose- and hormone-stimulated insulin secretion	23349487

S100a6	S100 calcium binding protein A6	+2.5	Controls the Ca^{2+}-dependent insulin release from the pancreatic beta cell	8119959
Aif1	Allograft inflammatory factor 1	+2.3	Present in the pancreas of prediabetic BB rats; alters glucose-stimulated insulin secretion	9391121
Myc	Myelocytomatosis oncogene	+2.3	Overexpression of c-Myc in isolated pancreatic islets suppresses insulin gene transcription and glucose-stimulated insulin secretion; transgenic mice overexpressing c-Myc in beta cells develop neonatal diabetes	11799123<br>12031967
Ctgf	Connective tissue growth factor	+2.3	Promotes proliferation of developing beta cells	21876171
Apcs	Amyloid P component, serum	+2.0	Increased expression in islets of patients with T2D	14578294
Maf	v-Maf musculoaponeurotic fibrosarcoma oncogene homolog (avian)	+1.9	Activates basal expression of the glucagon gene	17901057
Fbp1	Fructose-1,6-bisphosphatase 1	+1.8	Regulates glucose-stimulated insulin secretion of mouse pancreatic beta cells	20719858
Itgb6	Integrin, beta 6	+1.8	GWAS association with T2D	20418489
Fbp2	Fructose-1,6-bisphosphatase 2	+1.8	Highly expressed in human and rat pancreatic islets; regulates glucose-stimulated insulin secretion in pancreatic beta cells	15047617<br>18039179
Enpp1	Ectonucleotide pyrophosphatase/phosphodiesterase 1	+1.7	Association with T2D by genome-wide familial linkage studies; increased expression in human pancreatic islets of individuals with T2D	18071025<br>16096055
Tspan4	Tetraspanin 4	+1.7	Increased expression in human pancreatic islets of individuals with T2D	16096055
Sel1l	Sel-1 suppressor of lin-12-like (C. elegans)	+1.6	Encodes an ER membrane protein that is highly expressed in the pancreatic islets; mice with heterozygous inactivation of one allele have reduced beta cell mass and develop high fat diet-induced hyperglycemia	21536682
Vps13c	Vacuolar protein sorting 13 homolog C (S. cerevisiae)	+1.6	Genetic variants at the VPS13C locus are associated with the 2 hour blood glucose after an oral glucose challenge	20081857<br>21789219
Txnip	Thioredoxin interacting protein	+1.6	Proapoptotic protein; *Txnip* deficiency inhibits the mitochondrial death pathway	19875615

			underlying beta cell glucotoxicity	
Pla2g4a	Phospholipase A2, group IVA (cytosolic, calcium-dependent)	+1.6	Participates in glucose-induced insulin secretion	9030192 21896929
Eif4ebp1	Eukaryotic translation initiation factor 4E binding protein 1	+1.6	Encodes 4E-BP1 involved in regulation of protein translation; its expression is increased in islets of diabetic mice and *Eif4ebp1* deletion accelerates beta cell loss and exacerbates hyperglycemia in mouse models of diabetes	18316032
Tlr2	Toll-like receptor 2	+1.5	Tlr2-deficient mice are protected against beta cell dysfunction induced by a high-fat diet	20407745
Csrp1	Cysteine and glycine-rich protein 1	+1.5	Increased expression in human pancreatic islets of individuals with T2D	16096055
Agmo	Alkylglycerol monooxygenase	+1.5	Also known as TMEM195; GWAS associations with fasting glucose, indices of beta-cell function (HOMA-B) and T2D	20081858 20419449
Cartpt	CART prepropeptide	-2.3	CART regulates islet hormone secretion and is overexpressed in beta cells of T2D rats	16443761
Npy	Neuropeptide Y	-2.3	Insulin secretion is increased in pancreatic islets of *Npy*-deficient mice	17717054
Fev	FEV (ETS oncogene family)	-2.1	Mice lacking Fev (Pet1) have reduced insulin production and secretion and impaired glucose tolerance	22013016
Abcc8	ATP-binding cassette, subfamily C (CFTR/MRP), member 8	-2.0	Mutations in *ABCC8* result in permanent or transient neonatal diabetes mellitus, hyperinsulinemic hypoglycaemia familial, type 1 or are associated with T2D	7716548 8635661 16885549
Hmgn3	High mobility group nucleosomal binding domain 3	-1.9	Expressed in all pancreatic endocrine islet cells and controls insulin and glucagon levels; *Hmgn3*$^{-/-}$ mice have a mild diabetic phenotype	19651901 19885867
Chga	Chromogranin A (parathyroid secretory protein 1)	-1.8	*Chga*$^{-/-}$ mice have reduced islets size and decreased plasma insulin concentrations	18722481

Gipr	Gastric inhibitory polypeptide receptor	-1.7	GWAS association with the glucose and insulin responses to an oral glucose tolerance test; *Gipr*$^{-/-}$ mice have higher blood glucose levels with impaired initial insulin response after an oral glucose tolerance test	20081857<br>10611300
Pyy	Peptide YY (mapped)	-1.7	Ablation of *Pyy* in adult mice results in disruption of islet morphology and severe hyperglycaemia	22562022
Gpr119	G protein-coupled receptor 119	-1.7	Predominantly expressed in pancreatic polypeptide-secreting PP-cells; enhances the glucose-dependent insulin release	17070774<br>17289847
Glp1r	Glucagon-like peptide 1 receptor	-1.6	Binds the incretin GLP1, potentiating glucose-dependent insulin secretion from islet beta cells. *Glp1r*$^{-/-}$ mice show glucose intolerance and low levels of circulating insulin; selective restoration of GLP1R in islets of *Glp1r*$^{-/-}$ mice improves their glucose tolerance and pharmacological activation of GLP1R stimulates beta cell proliferation in mice	8898756<br>14966573<br>22182839
Plag1	Pleiomorphic adenoma gene 1	-1.6	*Plag1* overexpression leads to increased beta cell mass and hyperinsulinemia	20522588
Nkx2-2	NK2 homeobox 2	-1.6	*Nkx2-2*$^{-/-}$ mice develop diabetes due to arrested differentiation of pancreatic beta cells; essential for initiation of pancreatic beta cell differentiation as well as for maintenance of beta cell function in the mature islets; prevents beta to alpha cell reprogramming	9584121<br>14729487<br>17456846<br>22056672
Isl1	ISL LIM homeobox 1	-1.6	Required for the in the maturation, proliferation, and survival of the endocrine pancreatic cells; mediates the action of leptin on insulin secretion in mice	19502415<br>23504315
Mafb	v-Maf musculoaponeurotic fibrosarcoma oncogene homolog B (avian)	-1.5	Expressed in both alpha and beta cells during early development and only in alpha cells in adult life, regulating glucagon expression; Mafb-deficient mice show preferential reduction of beta cells.	16443760<br>18199433<br>20627934
