# Supplementary material for: Ageing is associated with molecular signatures of inflammation and type 2 diabetes in rat pancreatic islets
Source: Diabetologia. 2015 Dec 23;59:502–11. doi: 10.1007/s00125-015-3837-8 (PMC4742511; doi:10.1007/s00125-015-3837-8)
Supplement: Supplementary file 3 — (PDF 10 kb) [file 125_2015_3837_MOESM3_ESM.pdf]

ESM Table 3 DNA methylation levels at gene promoter regions in rat islets measured by bisulfite MassArray analysis

**Gene**	**3M (%)**	**15M (%)**	**p value**
Gene expression down-regulated with age			
Aurkb	2.2 (± 0.1)	3.1 (± 0.2)	**0.0087**
Gjb6	75.9 (± 2.9)	76.3 (± 1.9)	0.7879
Fam183b	9.5 (± 1.2)	10.9 (± 0.7)	0.5844
Cartpt	6.1 (± 0.3)	8.53 (± 0.27)	**0.0022**
Npy	2.1 (± 0.3)	3.3 (± 0.2)	**0.0152**
Cd79b	91.7 (± 2.1)	86.1 (± 1.1)	**0.0455**
Abcc8	4.3 (± 0.2)	5.6 (± 0.2)	**0.0043**
Gipr	12.4 (± 1.2)	16.1 (± 0.8)	**0.026**
Pyy	48.4 (± 2.6)	32.9 (± 0.8)	**0.0022**
Gpr119	12.0 (± 4.5)	40.3 (± 3.6)	**0.0087**
Gene expression up-regulated with age			
Cxcl9	72.0 (± 1.5)	59.8 (± 0.6)	**0.0022**
Spp1	71.8 (± 0.6)	62.2 (± 0.6)	**0.0022**
Reg3a	93.8 (± 0.7)	91.9 (± 0.5)	**0.0455**
Reg3b	2.4 (± 0.6)	14.6 (± 1.9)	**0.0022**
Tf	30.2 (± 0.9)	21.8 (± 1.1)	**0.0043**
Prss35	29.7 (± 1.9)	23.3 (± 0.6)	**0.0087**
Serpinb1a	60.3 (± 3.6)	50.2 (± 2.9)	0.1212
Col3a1	67.4 (± 3.2)	49.3 (± 2.3)	**0.0087**
C4b	68.1 (± 2.6)	41.9 (± 2.8)	**0.0022**
Dpt	78.4 (± 2.1)	61.1 (± 2.3)	**0.0022**
Aldob	16.7 (± 3.4)	18.5 (± 1.6)	0.5714
Ptf1a	10.2 (± 0.5)	9.4 (± 0.2)	0.303
Cldn10	63.5 (± 5.0)	77.7 (± 2.1)	**0.026**
Col1a1	37.4 (± 1.5)	33.4 (± 3.4)	0.1797
Gatm	4.8 (± 0.1)	5.0 (± 0.2)	0.329
Lgals3	2.9 (± 0.2)	2.9 (± 0.3)	0.7273

Mgst1	16.9 (± 1.8)	11.5 (± 0.2)	**0.0043**
Kcnq1	3.5 (± 0.6)	3.3 (± 0.5)	0.8983
Lgals1	35.6 (± 2.1)	26.9 (± 0.3)	**0.026**
Pah	86.8 (± 0.7)	56.5 (± 1.7)	**0.0022**
Fmo1	65.3 (± 1.9)	62.2 (± 2.5)	0.474
Il33	92.1 (± 0.8)	77.3 (± 3.3)	**0.0043**
Tspan8	20.2 (± 1.4)	18.2 (± 3.0)	0.974
Ptger3	8.2 (± 0.9)	9.3 (± 0.5)	0.5714
S100a6	23.5 (± 1.4)	21.8 (± 0.8)	0.3052
Ccdc80	1.6 (± 0.1)	2.8 (± 0.7)	0.3203
Ddah1	6.2 (± 0.4)	6.2 (± 0.4)	0.8939
S100a4	81.2 (± 1.6)	75.0 (± 1.0)	**0.0087**
Gbp2	55.2 (± 2.5)	53.1 (± 2.3)	0.5628
Lgals2	41.2 (± 2.9)	43.3 (± 9.1)	0.8745
Ctss	84.7 (± 1.5)	84.8 (± 0.3)	> 0.9999
Itgb6	19.4 (± 3.1)	25.4 (± 1.8)	0.0931
Fbp2	59.4 (± 5.0)	34.9 (± 2.6)	**0.0022**
Serping1	9.6 (± 0.9)	13.3 (± 0.3)	**0.026**
Tspan4	6.1 (± 0.2)	5.9 (± 0.6)	0.0931
Dmbt1	66.2 (± 3.2)	74.1 (± 1.1)	**0.026**
**LINE-1**	84.2 (± 0.5)	78.7 (± 0.5)	**0.0022**

Data represent mean values ± SEM (n=5-6 samples per group). P values were obtained by Mann-Whitney tests and significant differences are written in bold. Gene symbols written in bold and color are those that associate an absolute difference in methylation above 5% and p value <0.05 (red – loss of methylation with age; blue – gain of methylation with age).
