# Supplementary material for: Ageing is associated with molecular signatures of inflammation and type 2 diabetes in rat pancreatic islets
Source: Diabetologia. 2015 Dec 23;59:502–11. doi: 10.1007/s00125-015-3837-8 (PMC4742511; doi:10.1007/s00125-015-3837-8)
Supplement: Supplementary file 6 — (PDF 100 kb) [file 125_2015_3837_MOESM6_ESM.pdf]

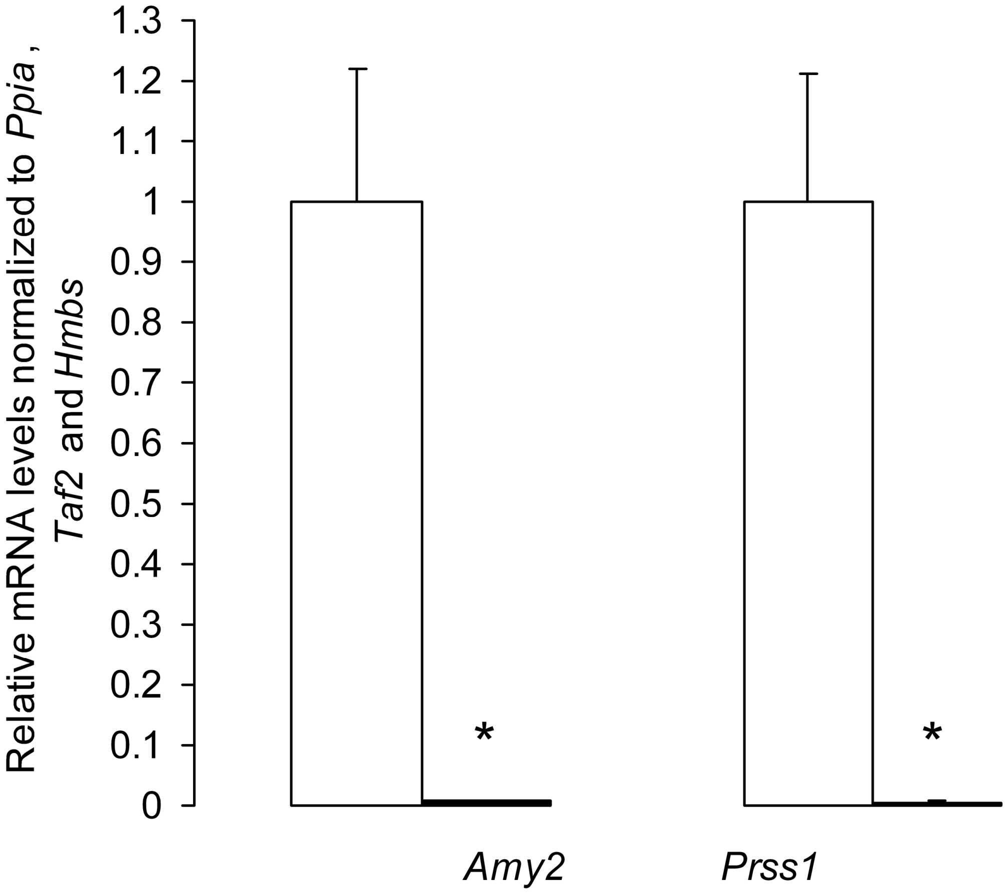

ESM Fig. 1 Assessment of islet sample purity. Expression levels of the acinar genes *Amy2* and *Prss1* were more than 100 fold lower in purified islets than in whole pancreas samples. White bars – whole pancreas, black bars – pancreatic islets. Data were obtained by quantitative real-time PCR (qRT-PCR) using 3 month (3M) old rats (n=3 samples in each group) and were normalized to the values obtained in the whole pancreas (arbitrarily set to 1).
