# Supplementary material for: Ageing is associated with molecular signatures of inflammation and type 2 diabetes in rat pancreatic islets
Source: Diabetologia. 2015 Dec 23;59:502–11. doi: 10.1007/s00125-015-3837-8 (PMC4742511; doi:10.1007/s00125-015-3837-8)
Supplement: Supplementary file 7 — (PDF 1212 kb) [file 125_2015_3837_MOESM7_ESM.pdf]

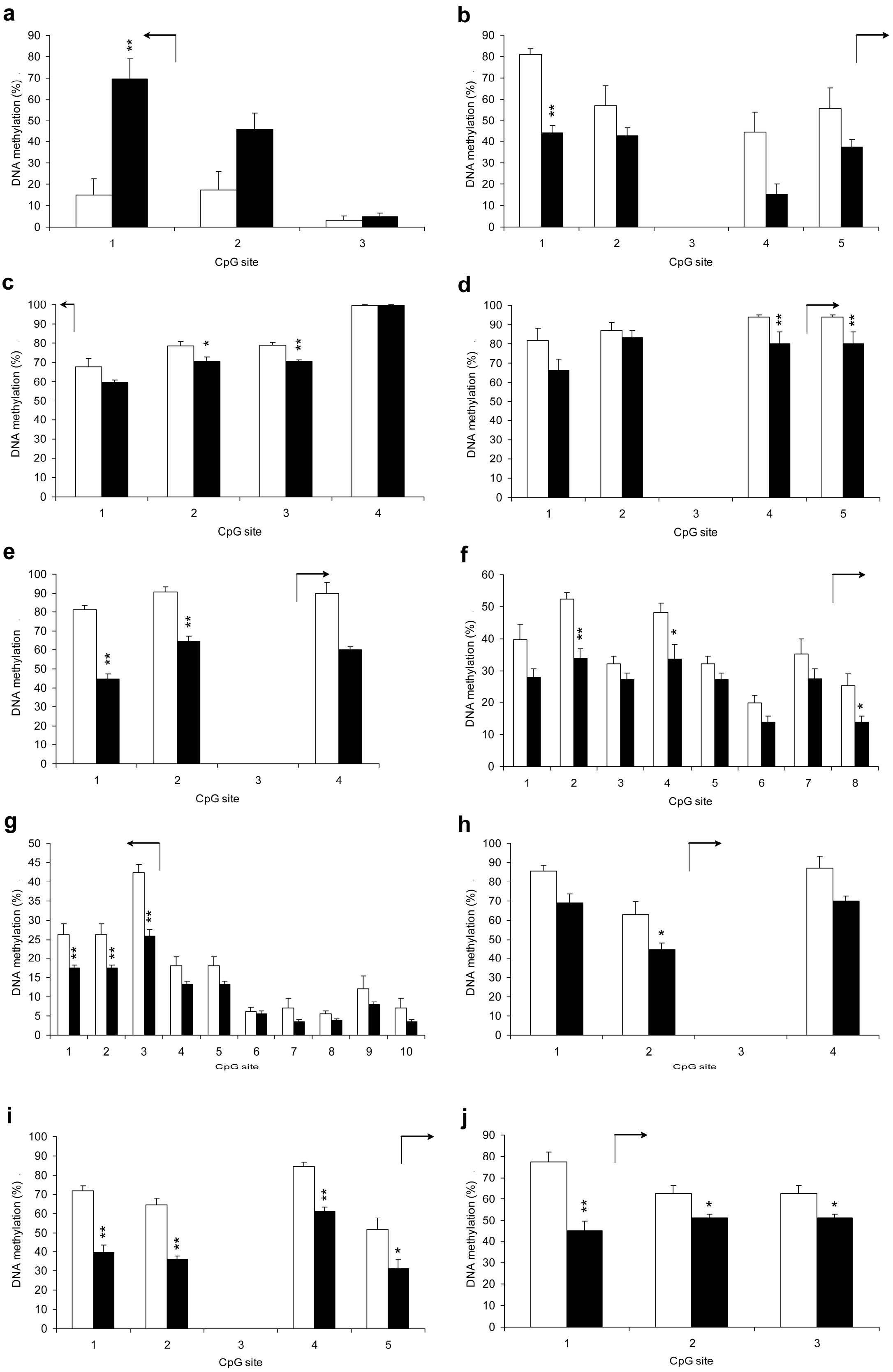
a
DNA methylation (%)
CpG site
**
b
DNA methylation (%)
CpG site
**
c
DNA methylation (%)
CpG site
*
**
d
DNA methylation (%)
CpG site
**
**
e
DNA methylation
CpG site
**
**
f
DNA methylation (%)
CpG site
**
*
*
g
DNA methylation (%)
CpG site
**
**
**
h
DNA methylation (%)
CpG site
*
i
DNA methylation (%)
CpG site
**
**
**
*
j
DNA methylation (%)
CpG site
**
*
*

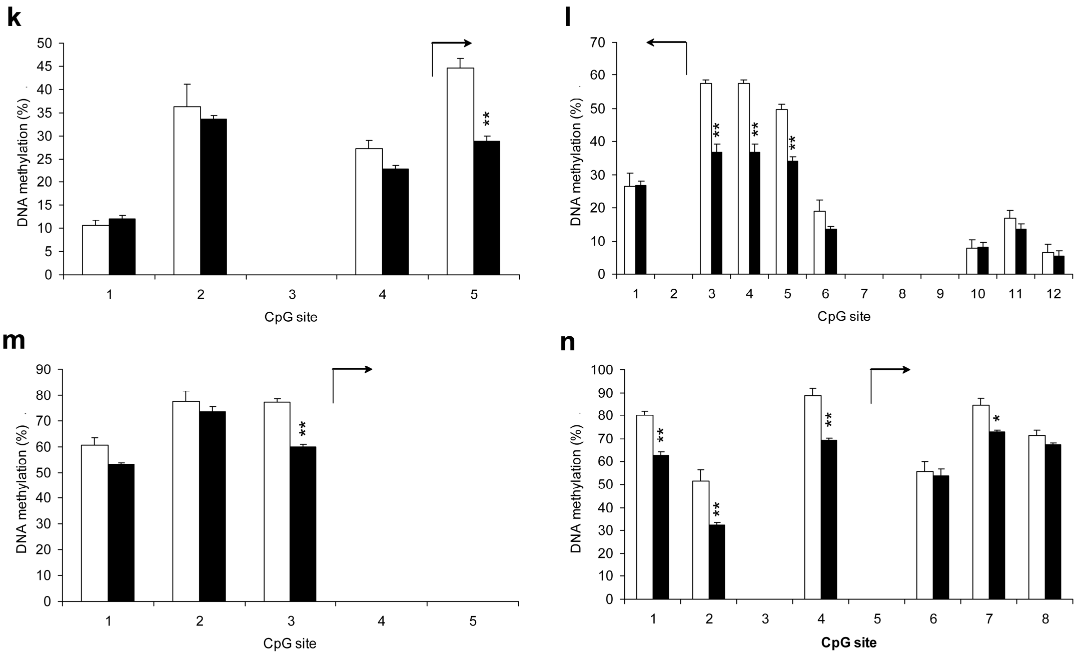

ESM Fig. 2 Bisulfite MassArray analysis of DNA methylation at promoter regions of genes with an absolute difference in methylation above 5% that demonstrated inverse relationship with mRNA expression levels: *Gpr119* (**a**), *Fbp2* (**b**), *S100a4* (**c**), *Il33* (**d**), *Pah* (**e**), *Lgals1* (**f**), *Mgst1* (**g**), *Dpt* (**h**), *C4b* (**i**), *Col3a1* (**j**), *Prss35* (**k**), *Tf* (**l**), *Spp1* (**m**) and *Cxcl9* (**n**). Data is shown per individual CpG dinucleotide; missing columns depict CpG dinucleotides contained within fragments with masses outside the range of 1,500-7,000 Da that cannot be analyzed by Sequenom. For all panels, white bars correspond to three months old (3M) samples and black bars to fifteen months old (15M) samples. Data represent the mean of n=5-6 samples; error bars represent SEM; * $p<0.05$; ** $p<0.01$; *** $p<0.001$ by Mann-Whitney tests; arrows indicate transcription start sites.
