# Supplementary material for: Ageing is associated with molecular signatures of inflammation and type 2 diabetes in rat pancreatic islets
Source: Diabetologia. 2015 Dec 23;59:502–11. doi: 10.1007/s00125-015-3837-8 (PMC4742511; doi:10.1007/s00125-015-3837-8)
Supplement: Supplementary file 8 — (PDF 280 kb) [file 125_2015_3837_MOESM8_ESM.pdf]

a

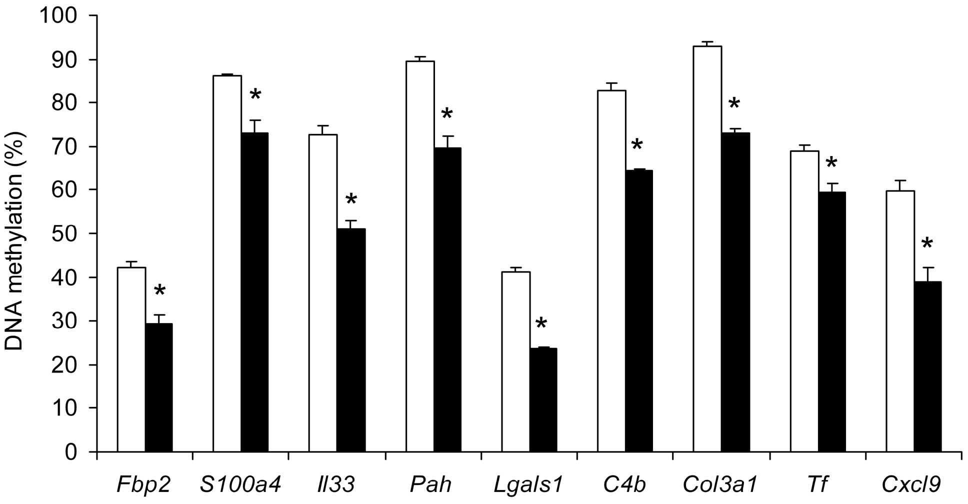

b

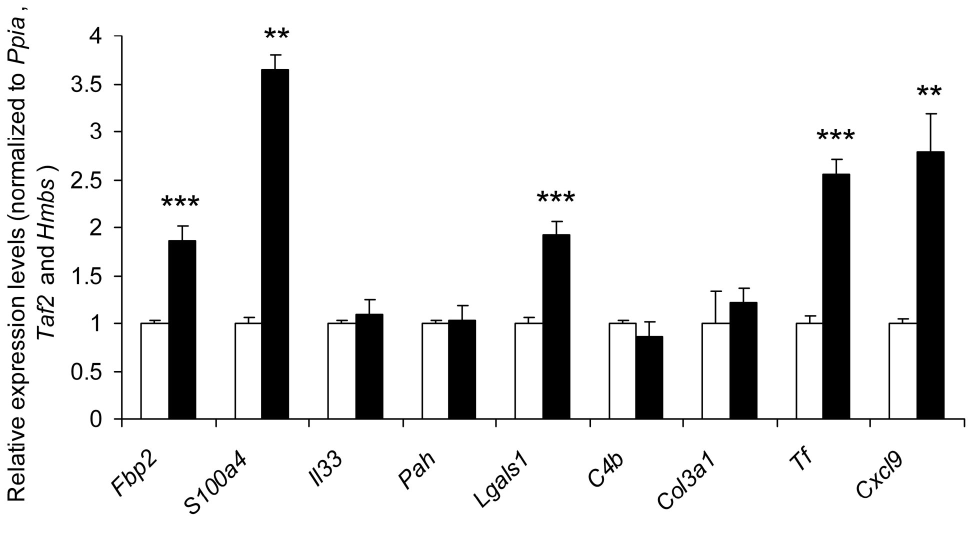

ESM Fig. 3 Zebularine treatment in BRIN-BD11 cells. (**a**) Bisulfite MassArray analysis of DNA methylation levels in cells exposed to vehicle (dimethyl sulfoxide – DMSO) or to 100 μM zebularine. Data represents the mean of n=4 samples per group. (**b**) Expression analysis by quantitative real-time PCR (qRT-PCR). Data represents the mean of n=6 samples per group. For both panels black bars correspond to BRIN-BD11 cells treated with 100 μM zebularine and white bars to control cells treated with vehicle (DMSO) only. Data represents mean values; error bars represent SEM; * $p<0.05$; ** $p<0.01$; *** $p<0.001$ by Mann-Whitney tests for panel (**a**) and obtained by using REST 2009 for panel (**b**).
