# Supplementary material for: Ageing is associated with molecular signatures of inflammation and type 2 diabetes in rat pancreatic islets
Source: Diabetologia. 2015 Dec 23;59:502–11. doi: 10.1007/s00125-015-3837-8 (PMC4742511; doi:10.1007/s00125-015-3837-8)
Supplement: Supplementary file 9 — (PDF 631 kb) [file 125_2015_3837_MOESM9_ESM.pdf]

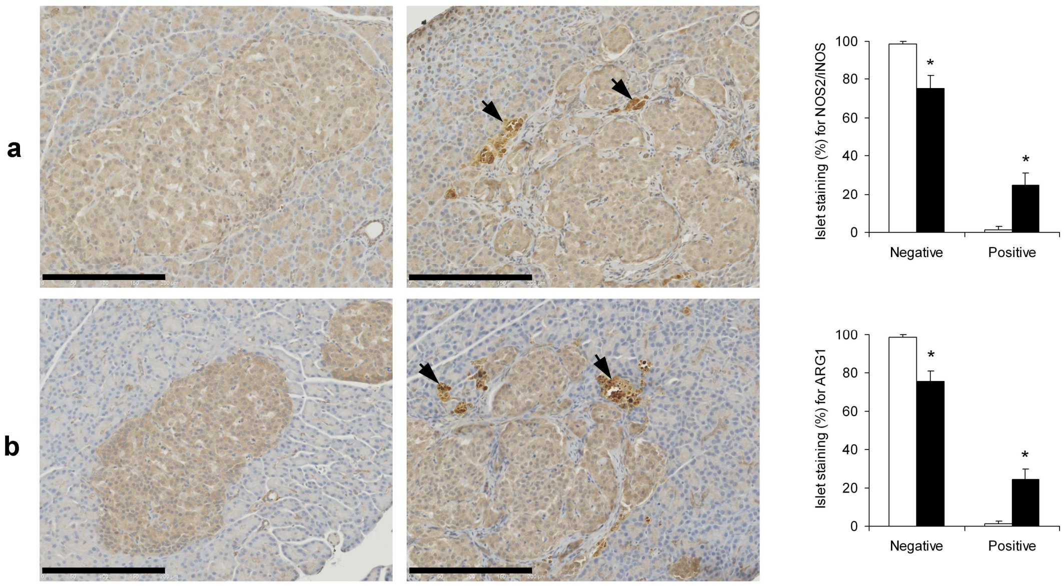

ESM Fig. 4 Immunohistochemical analysis of M1 and M2 macrophages in three months old (3M) and fifteen months old (15M) rat pancreata. (**a**) Immunostaining for nitric oxide synthase 2 – NOS2 (also denoted as iNOS), a marker of the "classically activated", pro-inflammatory M1 macrophages. (**b**) Immunostaining for arginase 1 – ARG1, a marker for the "alternatively-activated" anti-inflammatory M2 macrophages. For both panels the two types of macrophages are identified by strong staining against weaker staining in other cell types (arrows). Scale bars correspond to 200 μm. The histograms on the right side indicate percentages of pancreatic islets infiltrated by the corresponding types of macrophages analyzed. Data represent the means of n=4 samples per group; error bars represent SEM; * $p<0.05$ using Mann-Whitney tests.
